# Morphological and topographical characteristics of posterior supernumerary molar teeth: An epidemiological study on 25,186 subjects

**DOI:** 10.4317/medoral.19775

**Published:** 2014-08-17

**Authors:** Michele Cassetta, Federica Altieri, Matteo Giansanti, Roberto Di-Giorgio, Sabrina Calasso

**Affiliations:** 1 DDS, PhD Assistant Professor, Department of Oral and Maxillofacial Sciences, School of Dentistry, “Sapienza” University of Rome, Italy; 2 DDS, DDS, DDS. Assistant Researcher, Department of Oral and Maxillofacial Sciences, School of Dentistry, “Sapienza” University of Rome, Italy; 3 MD. Associate Professor, Department of Oral and Maxillofacial Sciences, School of Dentistry, “Sapienza” University of Rome, Italy

## Abstract

Objectives: To investigate the prevalence , gender difference , arch , morphology and position within the arch of supernumerary molar (SM) teeth in a referred Italian Caucasian population. Study Design: Records of 25,186 young patients were evaluated. Only data related to supernumerary teeth in the posterior region of the jaws were analyzed. The diagnosis of hyperdontia was formulated during the clinical and radiological examinations based on panoramic radiographs. Statistical analysis was conducted at level of subjects in the assessment of prevalence of SMs and sex ratio. Statistical analysis was conducted at level of teeth according to their morphological and topographic characteristics. The analysis of association between supernumerary morphology and arch, between supernumerary position and arch and between morphology and position was performed using the χ2 test (*P*≤ 0.05). Results: 61 posterior supernumerary teeth were found in 45 patients. The male to female ratio was 2.5:1 ;the mean age was 21.23 (IC:95%).The SMs were found more frequently in the maxilla (62.3%) than in the mandible; supernumerary teeth (60.7%) were more frequent than supplemental teeth. The SMs were mostly of tuberculate shape (56.8%) and paramolars teeth (64.9%) were more common than distomolars. 54% of teeth were erupted in the arch. No statistically significant relationship were found between the supernumerary teeth shape and the arch (*P*= 0.087) , between supernumerary teeth position and the arch (*P*=0.511) and between morphology and position (*P*=0.216). Conclusions: Epidemiological studies related to supernumerary teeth can be useful to clinicians in the early diagnosis of this anomaly. In this retrospective study the prevalence of SMs was 0.18%. SMs were more frequent in males and in the maxilla. Supernumerary were more frequent than supplemental; the conical morphology and paramolar position were the most common shape and position.

** Key words:**Hyperdontia, prevalence, supernumerary molars, distomolar, paramolar.

## Introduction

Supernumerary teeth (ST) can be defined as any teeth or tooth substance in excess of the usual configuration of the normal number of deciduous or permanent teeth (1-4). The condition is also known as hyperdontia (1). ST may occur singly, multiply, unilaterally or bilaterally, and in one or both jaws (2,3). Hyperdontia can be classified as “true” if there is a real increase in the number of teeth or as “false” if there is a deciduous tooth in the arch (5).

Although several theories have been suggested to explain their development, the precise etiology of these teeth is uncertain; current knowledge suggests that tooth anomalies result from a complex interplay of genetic factors and developmental processes (2,6). Hyperdontia is ascribed either to hyperactivity or to additional splitting off the dental lamina (2,3,6).

The prevalence of ST ranges between 0.1% and 4%, depending on the literature sources (7-9), and it is more frequent in males than in females with a male to female ratio (M:F) of 2:1 (2-4,10-15).

In the permanent dentition although ST can be encountered in any region of the dental arch, they are commonly located on the maxillary midline. This position is followed in decreasing order of frequency by maxillary fourth molars (which are located distally to the third molars), maxillary lateral incisors, mandibular fourth molars, and mandibular central incisors (4,6). Upper premolars are exceptional, as are upper and lower canines and lower lateral incisors (6).

In the deciduos dentition, ST are mostly encountered in the upper incisor region (16).

ST can be classified both morphologically and topographically (14,16,17).

A supernumerary tooth is a tooth of anomalous size and shape, and it not resembles a tooth with which it is associated (1,2). A supplemental tooth is instead a normally shaped tooth generally properly aligned in the arch (1,2).

ST may be classified topographically according to their position in the dental arch as mesiodens, paramolars (PMs), distomolars (DMs) (16). Briefly, supernumerary teeth that occur between the central incisors are referred to as mesiodens teeth, those situated lingually or bucally to a molar tooth are called paramolar teeth, those specifically located distally to the third molar as an accessory fourth molar are termed distomolar teeth (6,18); the other teeth are named according to the region where they are situated (6).

ST are further referred to as conical, tuberculate and infundibular in shape (14,16). Most frequently, supernumerary permanent teeth are conical in shape. They mostly present as mesiodens. The tuberculate form usually exceeds the size of the conical type, displays more than one cusp and is barrel-shaped. The root is mostly incomplete or missing entirely. Eruption into the oral cavity occurs only rarely. The infundibular teeth appear always in the upper incisor area, they have a crown with a typical introversion that gives it a funnel; their root is single and tapered.

In the deciduos dentition are more common supplemental teeth, whereas in the permanent dentition prevail supernumerary teeth in conical shape followed by supplemental teeth, supernumerary teeth in tuberculate and infundibular shape (6,11,15,18).

Multiple hyperdontia can be associated with Gardner syndrome, cleidocranial dysplasia, Fabry-Anderson syndrome, Ehlers-Danlos syndrome (7,18).

ST may erupt normally, be inverted or transverse, assume an ectopic position, or follow an abnormal path of eruption. ST can be diagnosed during a routine, clinical, or radiographic evaluation and sometimes they are not responsible for any discernible side effects on the neighboring teeth.

However, they can cause a variety of complications including delayed eruption (1,4), non eruption, crowding, or displacement (including rotations of permanent teeth) and, less frequently, development of odontogenic cysts or resorption of neighboring teeth (2,10,12,17).

The therapeutic approach is conditioned by the position of ST (erupted in the arch, erupted outside arch, impacted ) and the existence of pre-existent pathological disorders.

The aim of this study was to investigate the prevalence, gender differences, arch, morphology and position within the arch of supernumerary molar teeth in Italian Caucasian population.

## Material and Methods

In this retrospective study conducted at the Department of Oral and Maxillofacial Sciences of “Sapienza” University of Rome, a total of 25,186 subjects were analyzed between January 2006 and January 2013. Supernumerary molar teeth were diagnosed during clinical and radiological examinations based on panoramic radiographs. Only data relating to supernumerary teeth in the posterior region of the jaws were analyzed.

Panoramic radiographs evaluations and clinical examination were made by the same physician (M.C.)

The inclusion criteria were:

• Italian Caucasian subjects.

• Subjects in permanent dentition with at least a supernumerary teeth.

• Availability of a panoramic X-Ray.

Subjects with history of extractions of supernumerary teeth or maxillofacial anomalies such as cleft lip and palate, diseases associated with systemic conditions and syndromes such as cleidocranial dysplasia and Gardner syndrome were not included in this study.

The demographic variables (age and gender), SMs prevalence, morphology and position in the arch were evaluated.

Supernumerary molar teeth were divided according to morphological criteria in supplemental and supernumerary teeth.

Supernumerary teeth were divided in conical, tuberculate and infundibular in shape; whereas according to topographic criteria they were divided in paramolar and distomolar teeth (6,11,14,15,18).

The local ethical committee was informed about the study and the Helsinki Declaration protocols were followed.

- Statistical analysis

Statistical descriptive analysis was performed and data were analyzed using SPSS software (Statistical Package for the Social Sciences, IBM Corporation, New York, NY). The statistical analysis was conducted at level of subjects in the assessment of prevalence of SMs, sex ratio and age.

The statistical analysis was conducted at level of teeth according to their position and morphology. The analysis of association between supernumerary morphology and arch, between supernumerary position and arch and between morphology and position was performed using the χ2 test, which was assumed to be significant when the p-value was not greater than 0.05 (*P*≤ 0.05).

## Results

The prevalence was 0.18% (IC:95%). From the study sample, 45 subjects were found with 61 supernumerary molar teeth (32 males and 13 females; sex ratio 2.5:1). The mean age was 21.23 (IC:95%) (range: 17-35 years).

The distribution of the sample according to the jaw showed a higher prevalence in the maxilla (62.3%) than in the mandible ( Table 1). Regarding the morphological aspects, 24 of 61 (39.3%) were supplementary teeth, 18 were located in the lower jaw and 6 in the upper jaw; whereas 37 of 61 teeth (60.7%) were supernumerary teeth, 5 in the lower jaw and 32 in the upper jaw ( Table 1).

**Table 1:**
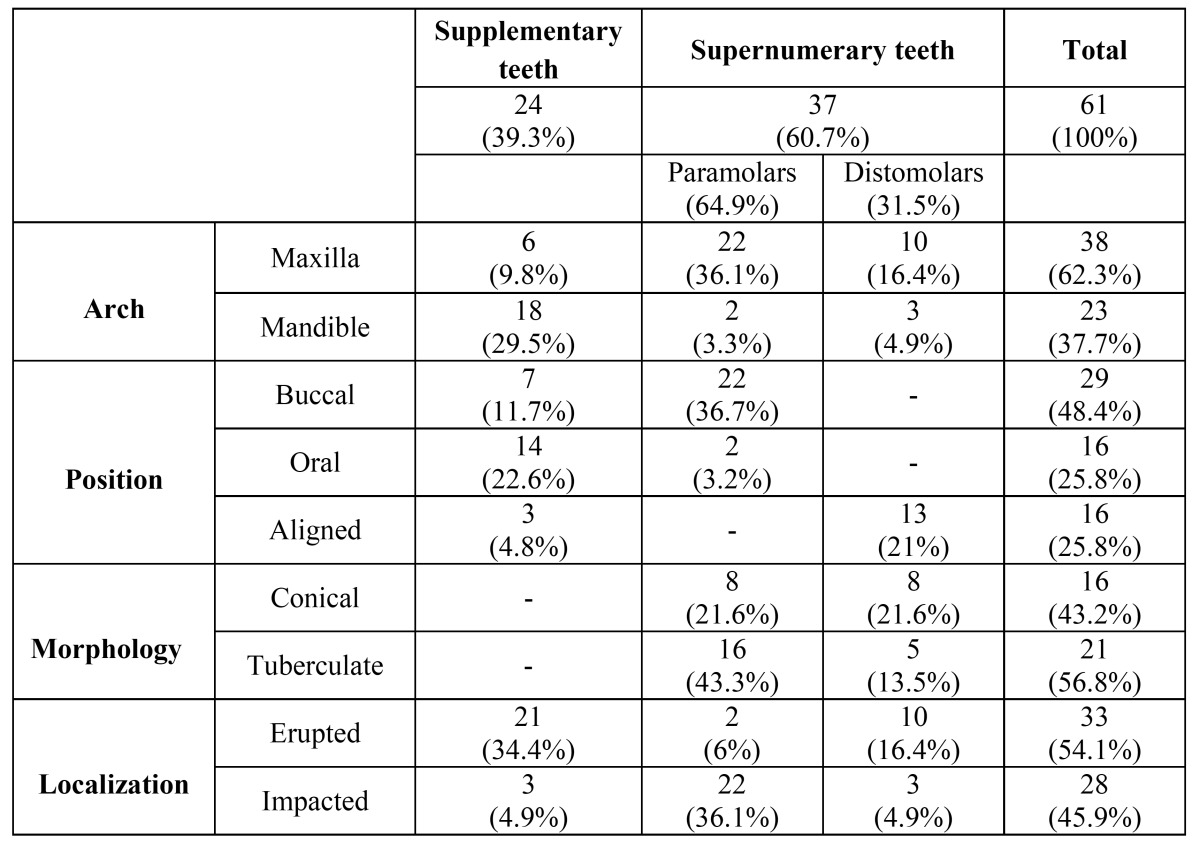
Distribution of supernumerary molar teeth according to arch, position, morphology and localization.

Concerning the 37 supernumerary teeth, 21 were tuberculate in shape, 18 in the upper jaw and 3 in the lower jaw; the other 16 teeth were conical in shape, 14 in the maxilla and 2 in the mandible. According to topographic criteria, 24 paramolar teeth and 13 distomolar teeth were found ( Table 1)

In the upper arch paramolars teeth (n=22) were more frequent than distomolars teeth (n=10), whereas in the lower arch distomolar were more frequent (n=3) ( Table 1).

When both morphological and topographic criteria were employed, distomolars teeth, mostly conical in shape, were found in the maxilla (n=8); whereas paramolars teeth were mostly tuberculate in shape (n=16) ( Table 1).

In the mandible all 3 distomolars were tuberculate in shape and all 2 paramolars teeth were conical in shape.

According to the position, all 13 distomolars were aligned in the arch ( Table 1); 22 of 24 paramolars teeth were positioned buccal to the arch; the 2 remaining paramolars teeth were positioned orally to the arch ( Table 1).

According to the position of supplemental teeth, all upper supplementary teeth (n=6) were buccal; in the lower jaw 14 of 18 supplementary teeth were in the oral side; 3 were aligned in the arch and one in the buccal side ( Table 1).

33 of 61 posterior supernumerary teeth (54.1%) were erupted into the oral cavity, while 28 (45.9%) were bone impacted ( Table 1).

The clinical exam was essential to diagnose lower supplemental teeth; whereas lower distomolars teeth were diagnosed mostly with the x-ray exam.

No statistically significant relationship were found between the supernumerary teeth shape and the arch (*P*= 0.087), between supernumerary teeth location and the arch (*P*=0.511) and between morphology and position (*P*=0.216) ( Table 2).

**Table 2:**
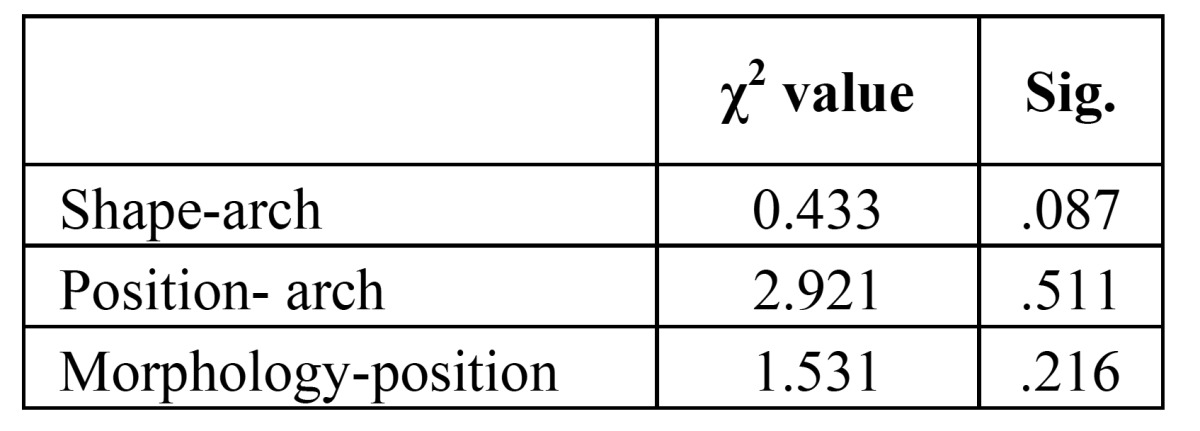
χ2 test between supernumerary morphology and arch , supernumerary position and arch and supernumerary morphology and position. Significance was set at *P*≤ 0.05.

## Discussion

Supernumerary teeth frequently occur in subjects with other dental anomalies and developmental disorders , this may confirm the importance of hereditary components or environmental factors (2,5).

ST are more frequent in permanent dentition but also can occur in deciduous dentition. Mason et al. reported that the prevalence of supernumerary teeth was 1.5%-3.5% in the permanent dentition and 0.3%-0.8% in the primary dentition (7). Concerning the prevalence of SMs Kara *et al*. (18) observed a prevalence of supernumerary molars of 0.33%, which is close to the findings of this study (0.18%).

In the present study, SMs were found in 32 males and 13 females, with a sex ratio of 2.5:1. The gender distribution reported by previous studies showed males being more commonly affected by supernumerary teeth in the permanent dentition (2-4,10-15). This finding, although relating to supernumerary teeth in the posterior region of the jaws, can confirm a clear male predilection of this pathology. The higher prevalence in males may be due to the association of supernumerary teeth with the autosomal recessive gene, which has a greater penetration in males (14).

In this study, a larger proportion of SMs was found in the maxilla (62.3%) than in the mandible (37.7%), giving a ratio of 1.7:1. This finding was in accordance with other authors (10,17,19). Grimanis *et al*. reported percentages of 79% of SMs affecting the maxilla (10). Menardía-Pejuan *et al*. reported percentages of 86.8% of SMs affecting the maxilla (17); Kara *et al*. reported that the frequency of SMs was much greater in the maxilla than in the mandible, with a ratio of 7:1 (18).

According to topographic criteria, in agreement with the literature (15,18), more paramolar teeth (n=24) than distomolar teeth (n=13) were found. Öztas *et al*. found 63% of paramolar teeth and 15.3% of distomolar teeth affected the posterior region (18). Also Montenegro *et al*. reported a low percentage of distomolar teeth (14.7%) in the molar region (15).

As found by other authors, supplementary teeth were more frequent in the lower jaw (18).

Considering the shape, three different types of supernumerary teeth have been described: conical, tuberculate, and infundibular teeth. In the permanent dentition supernumerary teeth appears generally conical (18), but in our results the tubercolate shape was the most common, this was probably due to the influence of the study design limited to supernumerary molar teeth.

The type of supernumerary teeth may cause various possible effects on the adjacent dentition (2,4,10,12,17,19,20).

Both upper paramolars and lower distomolar teeth were found impacted ; this result can be explained by the frequent tuberculate morphology: a big coronal portion combined to the absence of adequate space in the arch can determine teeth impaction (19). Foster and Taylor reported that tuberculate types of ST more frequently caused delayed eruption , whereas conical types more frequently caused displacement of the adjacent dentition (19). In the current study the panoramic x-ray examination has been a fundamental tool to diagnose impacted SMs.

Statistically significant relationship were not found between morphology and position (*P*=0.216); the majority of upper distomolar teeth were conical in shape (8 of 10 teeth); whereas upper paramolar teeth were mostly tuberculate (16 of 22 teeth). In the lower arch distomolars were mainly tuberculate (3 of 3 teeth) and paramolars were conical (2 of 2 teeth) ( Table 1).

## Conclusions

Epidemiological studies related to supernumerary teeth can be useful to clinicians in the early diagnosis of this anomaly.

On the basis of this retrospective study the prevalence of SMs in Italian Caucasian population was 0.18%.

SMs were more frequent in males than females (sex ratio 2.5:1); maxilla was more involved than mandible; supernumerary were more frequent than supplemental; the conical morphology and paramolar position were the most common shape and position.

No statistically significant relationship were found between the supernumerary teeth shape and arch (*P*= 0.087), between supernumerary teeth position and arch (*P*=0.511) and between morphology and position (*P*=0.216).
